# A Comparison of *DSM*-IV and *DSM*-5 Panel Members' Financial Associations with Industry: A Pernicious Problem Persists

**DOI:** 10.1371/journal.pmed.1001190

**Published:** 2012-03-13

**Authors:** Lisa Cosgrove, Sheldon Krimsky

**Affiliations:** 1 Edmond J. Safra Center for Ethics, Harvard University, Cambridge, Massachusetts, United States of America; 2 Department of Counseling Psychology, University of Massachusetts, Boston, Massachusetts, United States of America; 3 Department of Urban and Environmental Policy and Planning, and Department of Public Health and Community Medicine, Tufts University, Medford, Massachusetts, United States of America

## Abstract

Lisa Cosgrove and Sheldon Krimsky examine the new competing interest disclosure policy of the American Psychiatric Association (APA) and report that DSM panel members still have considerable financial conflicts of interest.

**Summary Points**

- The American Psychiatric Association (APA) instituted a financial conflict of interest disclosure policy for the 5th edition of the *Diagnostic and Statistical Manual of Mental Disorders* (*DSM*).
- The new disclosure policy has not been accompanied by a reduction in the financial conflicts of interest of *DSM* panel members.
- Transparency alone cannot mitigate the potential for bias and is an insufficient solution for protecting the integrity of the revision process.
- Gaps in APA's disclosure policy are identified and recommendations for more stringent safeguards are offered.

## Introduction

All medical subspecialties have been subject to increased scrutiny about the ways by which their financial associations with industry, such as pharmaceutical companies, may influence, or give the appearance of influencing, recommendations in review articles [1] and clinical practice guidelines [2]. Psychiatry has been at the epicenter of these concerns, in part because of high-profile cases involving ghostwriting [3],[4] and failure to report industry-related income [5], and studies highlighting conflicts of interest in promoting psychotropic drugs [6],[7]. The revised *Diagnostic and Statistical Manual of Mental Disorders* (*DSM*), scheduled for publication in May 2013 by the American Psychiatric Association (APA), has created a firestorm of controversy because of questions about undue industry influence. Some have questioned whether the inclusion of new disorders (e.g., Attenuated Psychotic Risk Syndrome) and widening of the boundaries of current disorders (e.g., Adjustment Disorder Related to Bereavement) reflects corporate interests [8],[9]. These concerns have been raised because the nomenclature, criteria, and standardization of psychiatric disorders codified in the *DSM* have a large public impact in a diverse set of areas ranging from insurance claims to jurisprudence. Moreover, through its relationship to the International Classification of Diseases [10], the system used for classification by many countries around the world, the *DSM* has a global reach.

After receiving criticism that *DSM*-IV had no financial disclosure of panel members, to its credit the APA instituted a mandatory disclosure policy [11]. The *DSM*-5 panel members are required to file financial disclosure statements, which are expected to be listed in the publication, and the APA has made a commitment to improve its management of financial conflicts of interest (FCOIs).

This new APA requirement makes the *DSM*'s disclosure policy more congruent with most leading medical journals and federal policies on FCOI. FCOIs are widely recognized as problematic because of the data showing a clear connection between funding source and study outcome whereby results are favorably biased toward the interests of the funder [12]–[14]—what has been referred to as the “funding effect” [15]. Some have argued that greater transparency of financial interests may facilitate a decline in FCOIs and a decrease in the potential bias that accompanies them, and that it may encourage professionals and consumers to more critically evaluate medical information [16]. Others are not sure that disclosure will reduce FCOIs and the potential for bias, because transparency alone just “shifts the problem from one of ‘secrecy of bias’ to ‘openness of bias’” [15]. Additionally, there is the concern that disclosure may open the door for subterfuge [17]. That is, when researchers or panel members list every affiliation that they have ever had, including funding from federal agencies, it can create a “signal-to-noise problem,” thereby obscuring the truth about deeply problematic financial relationships with industry.

We have reported elsewhere on industry relationships with *DSM*-5 task force members [18]. Although the composition of the task force has changed slightly since its formation in 2007 (e.g., Pilecki et al. [19] found 72% of the members had ties in early 2011) industry relationships persist despite increased transparency. Currently, 69% of the *DSM*-5 task force members report having ties to the pharmaceutical industry. This represents a relative increase of 21% over the proportion of *DSM*-IV task force members with such ties (57% of *DSM*-IV task force members had ties). This finding is congruent with emerging data from fields outside of psychiatry suggesting that transparency of funding source alone is an insufficient solution for eliminating bias [20]–[23].

In 2006 we analyzed all *DSM*-IV panel members' financial associations with industry [24]. We have undertaken a similar analysis for *DSM*-5 panels, which allowed us to compare the proportions of *DSM*-IV and -5 panel members who have industry ties. There are 141 panel members on the 13 *DSM*-5 panels and 29 task force members. The members of these 13 panels are responsible for revisions to diagnostic categories and for inclusion of new disorders within a diagnostic category.

**Figure 1:**
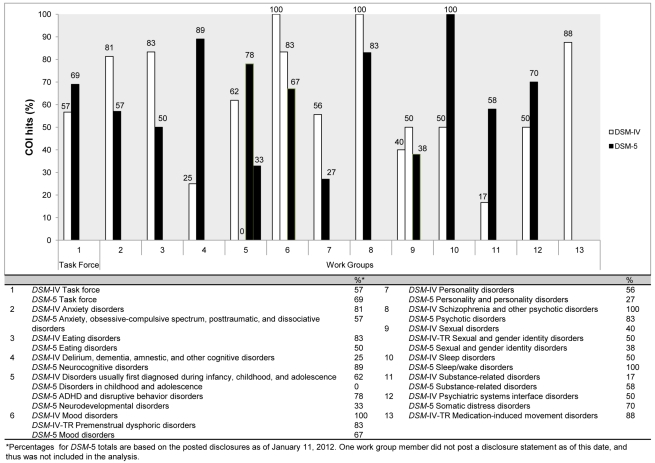
Comparison of financial conflicts of interest among *DSM*-IV and *DSM*-5 task force and work group members.

Three-fourths of the work groups (Figure 1; [2],[4]–[6],[8],[10]–[12]) continue to have a majority of their members with financial ties to the pharmaceutical industry. It is also noteworthy that, as with the *DSM*-IV, the most conflicted panels are those for which pharmacological treatment is the first-line intervention. For example, 67% (N = 12) of the panel for Mood Disorders, 83% (N = 12) of the panel for Psychotic Disorders, and 100% (N = 7) of the Sleep/Wake Disorders (which now includes “Restless Leg Syndrome”) have ties to the pharmaceutical companies that manufacture the medications used to treat these disorders or to companies that service the pharmaceutical industry.

## Gaps in APA's Disclosure Policy

Although the APA has made the disclosure of FCOIs of *DSM* panel members more transparent, there are important gaps in the current policy that need to be addressed:

1. The current APA disclosure policy does not require panel members to specifically identify speakers' bureau membership but rather cloaks it under “honoraria.” (A speakers' bureau usually refers to an arrangement between a commercial entity or its agent whereby an individual is hired to give a presentation about the company's product. The company typically has the contractual right to create and/or control the content of the presentation.) Therefore, despite increased transparency, it remains unclear how many individuals participate on speakers bureaus, because panel members may simply list “honoraria.” None of the *DSM* panel members identified participation on a speakers bureau. When we did an internet search of the 141 panel members, we found that 15% had disclosed elsewhere that they were members of drug companies' speakers bureaus or advisory boards. These internet searches were conducted for sources published in the years 2006 (1 year before the task force was appointed) to 2011, a time period congruent with published research on financial conflicts of interest. Searches included peer-reviewed articles, conferences, participation in continuing medical education events (i.e., courses and/or seminars for health professionals) and self-reporting of any industry ties following interviews with the media. Speakers bureau and advisory board participation were included in our analysis only when there was unambiguous information (e.g., “Dr. Smith discloses that he serves on the speakers bureau for Eli Lilly and Pfizer”) and both authors (LC, SK) were in agreement. The nature of these relationships needs to be spelled out more precisely; speakers bureau participation is usually prohibited elsewhere (e.g., for faculty in medical schools), as it is widely recognized to constitute a significant FCOI. Pharmaceutical companies refer to individuals who serve on speakers bureaus as “key opinion leaders” (KOLs) because they are seen as essential to the marketing of diseases as well as drugs.
2. Exclusions to the APA *DSM*-5 disclosure policy include unrestricted research grants [11]; that is, panel members are not required to disclose unrestricted research grants from industry. However, we would argue that this exclusion allows for commercial interests to be reflected in the revision process: there is no evidence to suggest that simply because money comes in the form of a large “unrestricted” research grant it does not create an obligation to reciprocate or invoke an implicit bias.
3. The current policy places high and arbitrary threshold limits on monies allowed from industry: *DSM* panel members are allowed to receive US$10,000 per year from industry (e.g., for consultancies), and panel members are allowed to have up to US$50,000 in stock holdings in pharmaceutical companies.
4. In contrast to other disclosure policies (e.g., the Physician Payments Sunshine Act of 2007 and the 2011 US National Institutes of Health policy on conflicts of interest), APA's policy does not require disclosure of the amount of money received from industry.

However, transparency alone cannot mitigate bias. Because industry relationships can create a “pro-industry habit of thought” [25], having financial ties to industry such as honoraria, consultation, or grant funding is as pernicious a problem as speaker's bureau participation. Over four decades of research from social psychology clearly demonstrates that gifts—even small ones—create obligations to reciprocate [26]–[28]. Also, because of the enormous influences of diagnostic and treatment guidelines, the standards for participation on a guideline development panel should be higher than those set for an average faculty member [29],[30].

## Conclusion

The *DSM*-5 will be published in about 14 months, enough time for the APA to institute important changes that would allow the organization to achieve its stated goal of a “… transparent process of development for the *DSM*, and …**an unbiased, evidence-based** ***DSM*** **, free from any conflicts of interest**” [emphasis added] [31]. Toward that goal we believe it is essential that:

1. As an eventual gold standard and because of their actual and perceived influence, all *DSM* task force members should be free of FCOIs.
2. Individuals who have participated on pharmaceutical companies' Speakers Bureaus should be prohibited from *DSM* panel membership.
3. There should be a rebuttable presumption of prohibiting FCOIs among the *DSM* work groups. When no independent individuals with the requisite expertise are available, individuals with associations to industry could consult to the *DSM* panels, but they would not have decision-making authority on revisions or inclusion of new disorders.

These changes would accommodate the participation of needed experts as well as provide more stringent safeguards to protect the revision process from either the reality of or the perception of undue industry influence.

## Abbreviations

APA: American Psychiatric Association
*DSM*: *Diagnostic and Statistical Manual of Mental Disorders*
FCOI: financial conflict of interest
KOL: key opinion leader
